# Testing the Drosophila *maternal haploid* gene for functional divergence and a role in hybrid incompatibility

**DOI:** 10.1093/g3journal/jkac177

**Published:** 2022-07-25

**Authors:** Dean M Castillo, Benjamin McCormick, Connor M Kean, Sahana Natesan, Daniel A Barbash

**Affiliations:** Institute of Agriculture and Natural Resources, University of Nebraska, Lincoln, NE 68588, USA; Department of Molecular Biology and Genetics, Cornell University, Ithaca, NY 14850, USA; Department of Molecular Biology and Genetics, Cornell University, Ithaca, NY 14850, USA; Department of Molecular Biology and Genetics, Cornell University, Ithaca, NY 14850, USA; Department of Molecular Biology and Genetics, Cornell University, Ithaca, NY 14850, USA; Department of Molecular Biology and Genetics, Cornell University, Ithaca, NY 14850, USA

**Keywords:** *Drosophila*, hybrid incompatibilities, speciation

## Abstract

Crosses between *Drosophila simulans* females and *Drosophila melanogaster* males produce viable F1 sons and poorly viable F1 daughters. Unlike most hybrid incompatibilities, this hybrid incompatibility violates Haldane’s rule, the observation that incompatibilities preferentially affect the heterogametic sex. Furthermore, it has a different genetic basis than hybrid lethality in the reciprocal cross, with the causal allele in *Drosophila melanogaster* being a large species-specific block of complex satellite DNA on its X chromosome known as the 359-bp satellite, rather than a protein-coding locus. The causal allele(s) in *Drosophila simulans* are unknown but likely involve maternally expressed genes or factors since the F1 females die during early embryogenesis. The *maternal haploid* (*mh*) gene is an intriguing candidate because it is expressed maternally and its protein product localizes to the 359-bp repeat. We found that this gene has diverged extensively between *Drosophila melanogaster* and *Drosophila simulans*. This observation led to the hypothesis that *Drosophila melanogaster mh* may have coevolved with the 359-bp repeat and that hybrid incompatibility thus results from the absence of a coevolved *mh* allele in *Drosophila simulans*. We tested for the functional divergence of *mh* by creating matched transformants of *Drosophila melanogaster* and *Drosophila simulans* orthologs in both *Drosophila melanogaster* and *Drosophila simulans* strains. Surprisingly, we find that *Drosophila simulans mh* fully complements the female sterile phenotype of *Drosophila melanogaster mh* mutations. Contrary to our hypothesis, we find no evidence that adding a *Drosophila melanogaster mh* gene to *Drosophila simulans* increases hybrid viability.

## Introduction

The evolution of reproductive isolation via hybrid incompatibilities can be complex, with multiple incompatibilities contributing to isolation within a single species pair. Two genetically distinct lethal hybrid incompatibilities exist between the sister species *Drosophila melanogaster* and *Drosophila simulans* (Sawamura, Watanabe, *et al.* 1993; Barbash 2010). When *D. melanogaster* females are crossed to *D. simulans* males, the F1 hybrid sons are invariably lethal, while the F1 daughters are generally fully viable, at least at lower temperatures (∼<25°C) (Sturtevant 1920; Barbash *et al.* 2000). Importantly, this pattern of lethality is not sex specific but rather caused by the presence of the *D. melanogaster* X chromosome. Experiments that can detect products of nondisjunction or use attached X chromosomes demonstrate that daughters inheriting both X chromosomes from their *D. melanogaster* mother are lethal while sons inheriting their X from their *D. simulans* father are viable (Barbash 2010). The lethality is caused by an incompatibility between the *D. melanogaster* allele of the gene *Hmr* on the *D. melanogaster* X, interacting with the *D. simulans* alleles of the autosomal genes *Lhr* and *GFZF* (Watanabe 1979; Hutter and Ashburner 1987; Brideau *et al.* 2006; Phadnis *et al.* 2015).

In contrast, the reciprocal cross of *D. simulans* females to *D. melanogaster* males produces viable F1 sons and poorly viable F1 daughters that die as early embryos (Sturtevant 1920). While the *D. melanogaster* X is again implicated in causing this embryonic lethality, Sawamura, Yamamoto, *et al.* (1993) showed that *Hmr* is not responsible for this F1 lethality. Instead, the lethal effect of the *D. melanogaster* X maps to the pericentromeric heterochromatin in a region called *Zhr* (Sawamura and Yamamoto 1993). The lethal effect of *Zhr^+^* appears to be caused by mis-segregation during early embryogenesis of a multimillion base pair block of complex satellite DNA sequences (Ferree and Barbash 2009). These satellite sequences are known alternatively as the 359-bp or the 1.688-g/cm^3^ satellites (Lohe and Brutlag 1986; Sawamura and Yamamoto 1993; Ferree and Barbash 2009). While *D. simulans* contains some dispersed 359-bp repeats, it does not have the large X-linked block found in *D. melanogaster* (Lohe *et al.* 1993; Lima *et al.* 2020; Sproul *et al.* 2020). This extensive difference in abundance of the 359-bp satellite between the species suggests that *D. melanogaster* may contain allele(s) that have co-evolved with the X-linked 359-bp satellite block to help promote its proper mitotic segregation.

This logic further implies that the lack of the X-linked 359-bp satellite block in *D. simulans* would cause it to be unable to regulate this satellite block when inherited from a *D. melanogaster* parent, leading to hybrid incompatibility. Because the F1 hybrid female lethality occurs during early embryonic development, the allele(s) hypothesized to be missing from *D. simulans* are likely to be maternally expressed (Sturtevant 1920; Ferree and Barbash 2009). The penetrance of F1 hybrid female lethality is highly variable across strains, which has complicated efforts to identify the genes causing this lethality (Gérard and Presgraves 2012). Sawamura, Taira, *et al.* (1993) identified a strain of *D. simulans* called *maternal hybrid rescue* (*mhr*) that produces high viability of F1 hybrid daughters and mapped the causal locus (or loci) to the second chromosome. Orr also implicated the *D. simulans* second chromosome in contributing to hybrid lethality in this cross (Orr 1996). Another study directly tested the satellite-binding protein D1 as a candidate but found no evidence for a role in the incompatibility (Ferree and Barbash 2009). Further attempts to identify the genetic basis of the *D. simulans* maternal effect on hybrid viability led to the plausible conclusion that it is a polygenic effect (Gérard and Presgraves 2012). Others have suggested that the incompatibility may be caused by the absence in *D. simulans* of maternally deposited small RNAs homologous to the 359-bp repeat, rather than by protein-coding genes (Ferree and Barbash 2007). Several studies have shown that such RNAs are produced by heterochromatic satellites including the X-linked 359-bp satellite block (Usakin *et al.* 2007; Wei *et al.* 2021), though their potential role in hybrid lethality remains untested.

The X-linked gene *maternal haploid* (*mh*) is an intriguing candidate for contributing to this interspecific incompatibility. Mutations in *mh* were first identified based on its female sterility phenotype (Gans *et al.* 1975). Embryos from *mh* mutant mothers typically arrest within the first few nuclear cycles with condensation defects specific to the paternally inherited chromosomes, with a minority reaching late embryogenesis as lethal gynogenetic haploids (Zalokar *et al.* 1975; Loppin *et al.* 2001). The *mh* gene encodes a predicted metalloprotease, homologs of which are involved in DNA damage repair (Delabaere *et al.* 2014; Tang *et al.* 2017). Most relevant to this study, the Mh protein localizes to the 359-bp satellite during embryogenesis and the satellite shows aberrant segregation in the embryonic progeny of *mh* mutant mothers (Tang *et al.* 2017). These findings, as well as the evolutionary patterns described below, motivated us to test for functional divergence of *mh* orthologs between *D. melanogaster* and *D. simulans* and for possible effects on hybrid lethality.

In particular, we tested the hypothesis that hybrid female embryonic lethality results from the inability of the maternally expressed *D. simulans* Mh protein to properly interact with the paternally inherited X-linked *D. melanogaster* 359-bp satellite block. Under this hypothesis, we predict that adding *mel-mh* to *D. simulans* mothers would suppress hybrid lethality.

## Materials and methods

### Nomenclature

We use the abbreviations *mel-mh* and *sim-mh* to refer to the *mh* ortholog in *D. melanogaster* and *D. simulans*, respectively. We use *phi{mel-mh-Gfp}* and *phi{sim-mh-Gfp}* to designate phiC31-mediated transgenes containing *Gfp* fusions of *mel-mh* and *sim-mh*, respectively. As described in the *Results*, *D. simulans* has a tandem duplication of *mh*. The *mh-p* copy is more similar than the *mh-d* copy in sequence and structure to *D. melanogaster mh*. We therefore consider *sim-mh-p* to be the ortholog of *D. melanogaster mh*, and for simplicity, refer to it as *sim-mh*.

### Drosophila stocks

*D. melanogaster* stocks *w mh^6^/FM7a, P{sChRFP}1* and *w, mh^31^/FM7, Gfp^+^* were kindly provided by Xiaona Tang and Yikang Rong. *D. simulans* stocks containing *attP* landing sites were kindly provided by David Stern (Stern *et al.* 2017).

### Sequence analysis

Genome sequences were obtained from *D. melanogaster* release 6 and the second-generation *D. simulans* release (Hu *et al*. 2013; Hoskins *et al*. 2015). The sequences for both duplicates of *mh* from the *D. simulans* genome were taken from the genome sequence and aligned with the *D. melanogaster* ortholog to determine the consensus coding sequence (see *Results*). We then calculated D_N_/D_S_ between the *D. melanogaster mh* and both *D. simulan*s orthologs using MEGA 11 (Tamura *et al.* 2021). We compared these ratios to a genome-wide sample of D_N_/D_S_ ratios from a previously published study to determine their percentile and relative rate of evolutionary change (Stanley and Kulathinal 2016). These data all consist of pairwise sequence comparisons between *D. melanogaster* and *D. simulans* orthologs. Iso-Seq sequences of mRNA transcripts from adult males were also analyzed to determine the reading frame and structure of the *mh* duplicates (Nouhaud, 2018). To do this, we ran a BLASTN search on the full length *mh-d* sequence, which, after manual filtering to remove duplicates and reads mapping to *mh-p* or other genes, turned up 4 unique reads mapping to *mh-d*. Out of these, only 2 reads mapped to *mh-d* upstream of exon 3, and neither of these reads mapped to potential exons 1 and 2.

### *mh* transgene constructs

The *mel-mh-Gfp* transgene was kindly provided by Xiaona Tang and Yikang Rong and previously described as *gfp-mh-pTV2gw* (Tang *et al.* 2017). We added an *attB* site to this to create the plasmid *gfp-mh-pTV2gw-attB* by PCR-amplifying using oligos 502/503 from a plasmid with an *attB* site that derived from the *pTA-attB* plasmid (Groth *et al.* 2004). The PCR product was digested using NotI and inserted into *gfp-mh-pTV2gw* at its NotI site. All oligonucleotide sequences are listed in Supplementary Table 1.

**Fig. 1.**
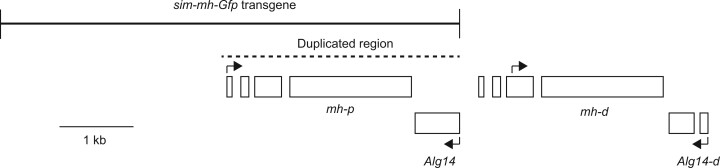
Map of *mh* region in *D. simulans* demonstrating the duplication event. The region in *D. melanogaster* has the same structure as the *mh-p* and *Alg14* regions. The dashed line shows the region duplicated to form *mh-d* and *Alg14-d*. Black arrows show translation start sites (Chakraborty *et al.* 2021). The 2 boxes shown for *Alg14-d* represent a frameshift relative to *Alg14*. A full annotation of this region is shown in Supp File 1.

The *w^+^-attB-sim-mh-eGfp* (p834) construct was designed to be parallel in structure to *gfp-mh-pTV2gw-attB* (Fig. 1 and Supplementary File 1) and was made in the following steps:

1. *w^+^-attB-sim-mh*. The *D. simulans mh* genomic region covering the coding region and ∼3000 bp upstream was PCR amplified from the strain *w^501^* using oligos 2099/2100 and cloned into pCR-Blunt II-TOPO (Invitrogen). The sequence of *sim-mh* was confirmed by Sanger sequencing (using oligos 788, 823, 2078, 2079, 2080, 2081, 2082, 2083, 2084, and 2101). The insert was then released by XbaI digestion and ligated into the XbaI site of the plasmid *w^+^-attB*, a gift from Jeff Sekelsky (Addgene plasmid # 30326; http://n2t.net/addgene:30326; RRID: Addgene_30326). The resulting clone *w^+^-attB-sim-mh* was confirmed by checking the pattern of restriction enzyme digestion.
2. *pCR-Blunt II-TOPO_sim-mh(partial)-eGfp*. The coding sequence of *eGfp* was inserted into the coding sequence of *sim-mh* immediately after the start codon by Gibson assembly of fragments termed Dsmh1, eGfp, and Dsmh2. Dsmh1 and Dsmh2 were PCR amplified from *D. simulans* (strain *w^501^*) using oligo pairs 2016/2017 and 2020/2021, respectively, while *eGfp* was amplified using oligos 2018/2019 from pEGfp-attB (Drosophila Genomics Resource Center). A fusion product of Dsmh1- eGfp -Dsmh2 was PCR-amplified from the Gibson Assembly reaction mixture, using oligos 2080/2082 and cloned into pCR-Blunt II-TOPO. The sequence of the insert, i.e. the fusion of eGfp CDS and Dsmh (partial), was checked by Sanger sequencing using oligos 498, 788, 823, and 2081.
3. *w^+^-attB-Dsmh-eGfp*. The Dsmh(partial)_eGfp fragment was released from the pCR-Blunt II TOPO vector and ligated into w^+^-attB-sim-mh using double digestion (BsiWI-BstEII). The final construct w-attB-sim_mh-eGfp was confirmed by checking the restriction pattern of the construct.

### *mh* transgenic lines

*D. melanogaster* transgenic lines were made by phiC31-mediated integration into the strain *y w P{nos-phiC31\int.NLS}X; P{CaryP}attP40*. *D. simulans* transformant lines were made by phiC31-mediated integration into the strains *y w; pBac{3XP3::EYFP, attP}1048-2R* and *y w; pBac{3XP3::EYFP, attP}1029-3R* (Stern *et al.* 2017). Microinjections were done by Rainbow Transgenic Flies, Inc.

### Fertility tests

The *mel-mh-Gfp* and *sim-mh-Gfp* transgenes transformed into *D. melanogaster* were compared for their ability to complement the female sterility of *mh* null mutations. A small-scale pilot experiment was initially done at room temperature by crossing single virgin *w mh^6^/w mh^31^; {mh-Gfp, w^+^}attP40/+* females to 2 DGRP-882 (wild-type) males, where *mh-Gfp* represents either of the 2 transgenes. Two sets were done: the first with females aged 3–4 days old before mating and the second aged 9–10 days. Vials were cleared after 5 days; if either the female or both males were dead, then the vial was discarded.

To generate F1 females to assay in a large-scale experiment, a parental cross was set up of *w mh^6^; {mh-Gfp, w^+^}attP40* females and *mh^31^/Y* males, where *mh-Gfp* represents either of the 2 transgenes. Virgin F1 daughters of genotype *w mh^6^/w mh^31^; {mh-Gfp, w^+^}attP40/+* were collected and aged for 3–5 days, followed by test crosses containing 1 virgin female and 2 Canton-S (wild type) males. After 4–5 days, parents were flipped to new vials and flipped again after another 4–5 days. At each flip, if either the female or both males were dead, then the vial was discarded. Otherwise, vials were kept for 16 (27°C) or 18 (25°C) days and all progeny counted. Three flips were performed at 27°C and 4 at 25°C; however, very few parents survived until the fourth flip at 25°C and, thus, we only report the first 3 flips in Fig. 3b. The overall time period across all 3 flips was 12 days at 25°C and 12–13 days at 27°C. Progeny per day are reported to normalize between flips that were 4 or 5 days.

### Hybrid viability tests

The *mel-mh-Gfp* and *sim-mh-Gfp* transgenes transformed into *D. simulans* were assessed for their ability to rescue female viability in F1 *D. simulans*/*D. melanogaster* hybrids as compared to a matched control group lacking the transgenes. To generate F1 hybrids either carrying or not carrying a *mh-Gfp* transgene, for each transgenic *D. simulans* line, the following parental crosses were set; first, *w^501^* virgin females were crossed to *y w/Y; {mh-Gfp, w^+^}* males, where *mh-Gfp* represents either of the 2 transgenes. Virgin daughters of the genotype *w^501^/y w; {mh-Gfp, w^+^}/+* collected from this cross were subsequently mated to *w^501^/Y* males. For each set, 30–40 *y? w/w^501^; {mh-Gfp, w^+^}/+* and *y? w/w^501^* virgin daughters were separately collected, aged 0–1 days, and mated to 40–50 3–5-day-old *D. melanogaster* Canton-S virgin males. The crosses were kept at room temperature (19.4–22.0°C) and flipped every 2–4 days until they stopped producing progeny; the adult F1 hybrids were scored for sex.

### Western blots

Young female virgin flies were fed yeast paste 2–3 days prior to dissection. Ovaries were dissected in 0.7% NaCl with 2 pairs of tweezers after flies were anesthetized by carbon dioxide. Ovaries were collected into 1.7-ml microcentrifuge tubes and flash-frozen in liquid nitrogen and then stored at −80°C until all dissections were completed.

To extract proteins, ovaries were ground in SDS sample buffer (62.5 mM Tris, pH 6.8, 2% SDS, 10% glycerol, 1% ß-mercaptoethanol, 0.05% bromophenol blue), boiled for 3 minutes, and centrifuged at 10,000 rpm for 5 min. Proteins were separated via 7.5% SDS-PAGE using a BioRad Mini-PROTEAN Vertical Electro Cell and transferred to polyvinylidene difluoride membrane using BioRad Mini Trans-Blot. The protein standard was Thermo Scientific PageRuler Prestained Protein Ladder, 10–180 kDa.

The protein-bound membrane was blocked in 5% skim milk in TBST (150 mM NaCl, 20 mM Tris, pH 7.5, 0.1% Tween-20) for 1 h at room temperature, followed by primary antibody incubation at 4°C for 16 h, and secondary antibody incubation at room temperature for 1 h. The membrane was washed 3 × 10 min in TBST after each antibody incubation. Primary antibodies used were: Anti-Gfp Rabbit Polyclonal Antibody (1/5,000, Rockland Immunochemical 600-401-215S) and Monoclonal Mouse Anti-α-Tubulin antibody (1/20,000, Sigma T9026), and secondary antibodies used were: HRP-Goat Anti-Rabbit IgG (H + L) (1/4,000, Jackson 111-035-003) and HRP-Goat Anti-Mouse IgG (H + L) (1/8,000, Jackson 115-035-003). Antibodies were diluted in either 5% skim milk or 5% BSA, in TBST. Signals were detected by applying ECL2 Western Blotting Substrate (Thermo Scientific 80197) to the membrane and exposing it to autoradiography film (VWR 490001-930).

## Results

### *mh* is duplicated in *D. simulans*

When attempting to identify the *mh* ortholog in *D. simulans*, we noticed 2 different regions with homology to *mel-mh*. The first region was a contig on the X with high similarity but that appeared to be incomplete and possibly contain partial duplications. While pursuing this analysis, a PacBio assembly of the *D. simulans* genome reported that *mh* is tandemly duplicated on the X along with the flanking gene *alg14* (Chakraborty *et al.* 2021). Following Chakraborty *et al.* (2021), we refer to the *D. simulans* duplicates as *mh-p* (mh-proximal) and *mh-d* (mh-distal), though as noted below we suggest that *mh-p* can also be considered the parental copy and *mh-d* the daughter copy (Fig. 1).

We confirmed the duplication structure of *D. simulans mh* in an independent assembly of *D. simulans* created using Nanopore sequencing (Miller *et al.* 2018). The Nanopore and PacBio assemblies fully agree in structure, with the exception of a ∼25-bp insertion in the PacBio assembly immediately distal to the proximal copy (Supplementary File 1). The 2 genome assemblies also contain no differences in the coding sequences of either *mh-p* or *mh-d*.

We used Artemis and the Artemis Comparison Tool to compare and annotate the structures of the *D. melanogaster* and *D. simulans mh* regions (Carver *et al*. 2008; Supplementary File 1). We estimate that the duplicated region corresponds to approximately 3181 bp (Fig. 1). This leaves *D. simulans mh-d* having a duplication of the *mh-p* 3′ region as its 5′ region, which may be responsible for its novel testis-enriched expression pattern reported by Chakraborty *et al.* (2021). There is a 16-bp deletion in the coding region of what would correspond to exon 1 of *mh-d*, relative to *mh-p* and *D. melanogaster mh*. We confirmed that *mh-d* has this deletion in 5 *D. simulans* strains that were sequenced using Sanger sequencing (Begun *et al.* 2007). This deletion could change the coding potential of exon 1, or alternatively the transcript could potentially splice to exon 2 and restore the same reading frame as in *mh-p*. However, using Iso-Seq reads from adult male *D. simulans* testes, we did not find evidence that the potential exon 1 or exon 2 are expressed (see *Materials and Methods*), consistent with the conclusions of Chakraborty *et al.* (2021). This suggests that *mh-d* produces a truncated product relative to *mh-p*, with the apparent transcription start site being ∼50–80-bp upstream of exon 3. We have annotated the *mh-d* CDS as beginning at the first ATG in exon 3, with potential exons 1 and 2 also indicated (Fig. 1 and Supplementary File 1).

Regardless of this uncertainty regarding the N-terminal structure of *mh-d*, *mh-p* has greater similarity *D. melanogaster mh* in its structure, primary sequence, and expression pattern, suggesting that *mh-p* can be considered to be the parental copy and *mh-d* the daughter copy of the duplication. We thus define *D. simulans mh-p* as the ortholog of *D. melanogaster mh*.

The second region of lesser homology mapped to an intron of the *tkv* gene on chr 2. This region of chr 2 is annotated as the pseudogene CR14033 and was identified as producing siRNAs in testis (Czech *et al.* 2008; Okamura *et al.* 2008) homologous to a region of *mh* (Czech *et al.* 2008). This pseudogene is present in multiple *Drosophila* species including *D. ananassae* and *D. pseudoobscura*, but the region homologous of CR14033 homologous to *mh* is only present in *D. melanogaster* and its sister species *D. simulans* and *D. sechellia* (Sperry 2016).

### *mh* coding sequences are rapidly evolving

We calculated pairwise divergence among the *mh* genes in *D. melanogaster* and *D. simulans* (Table 1). The D_N_/D_S_ ratios are relatively high compared to a genome-wide sample of loci, indicating substantial nonsynonymous divergence. The comparison between *mel-mh* and *sim-mh-p* had a D_N_/D_S_ ratio of 0.373, placing it in the top 10% of the genome-wide distribution. The comparison between *mel-mh* and *sim-mh-d* had a D_N_/D_S_ ratio of 0.509, which is in the top 5% of the genome distribution. For reference, the hybrid incompatibility loci *Hmr* and *Lhr* are in the top 3% of the distribution. D_N_/D_S_ is also higher between the paralogs *sim-mh-p* and *sim-mh-d* than between the orthologs *mel-mh* and *sim-mh-p*.

**Table 1.** Divergence of *mh* genes between *D. melanogaster* and *D. simulans*.

Gene 1	Gene 2	D_N_	D_S_	D_N_/D_S_	Percentile
*mel_mh*	*sim-mh-p*	0.056	0.150	0.373	91.2
*mel_mh*	*sim-mh-d*	0.086	0.168	0.509	95.3
*sim-mh-p*	*sim-mh-d*	0.040	0.066	0.605	n/a

The numbers of nonsynonymous and synonymous substitutions per site were estimated using the Nei-Gojobori method as implemented in MEGA 11. Percentile refers to rank of D_N_/D_S_ relative to 10,766 genes for which D_N_/D_S_ was compared between *D. melanogaster* and *D. simulans* (Stanley and Kulathinal 2016). Note that *mh* was not included in the Stanley and Kulathinal (2016) gene set because the *D. simulans* ortholog was not identified at that time.

### *D. simulans mh* complements *D. melanogaster mh* mutants

**Fig. 2.**
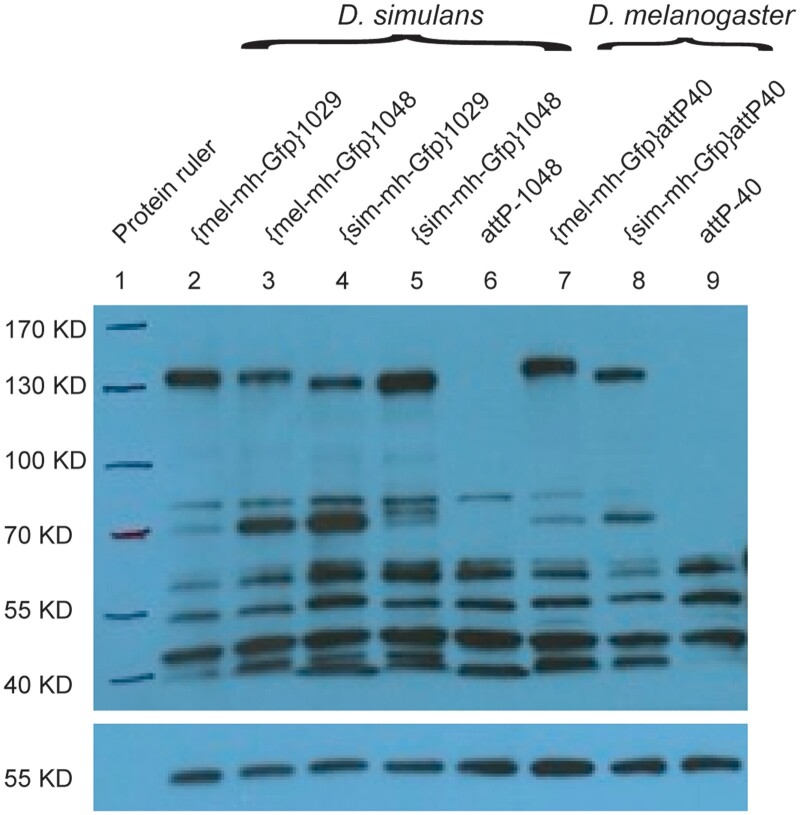
Western blot comparing Mh-Gfp protein accumulation in different transgenic genotypes. Western blot with an anti-Gfp antibody recognizes the Mh-eGfp protein in ovary extracts from females (top panel). Signals on the same membrane detected by an anti-α-Tubulin antibody served as a loading control (lower panel). Protein sizes of the prestained protein ladder were hand-marked on the membrane. Lanes 2–6 are from *D. simulans* extracts and lanes 7–9 from *D. melanogaster* extracts. Two independent transformants of the transgenes in *D. simulans* were analyzed, at *attP* sites 1029 and 1048. The *attP-1048* and *attP-40* samples are negative controls because they are the untransformed strains that carry the *attP* sites. The predicted molecular weights of mel-Mh-Gfp and sim-Mh-Gfp proteins are 108.6 and 109 kDa, respectively.

**Fig. 3.**
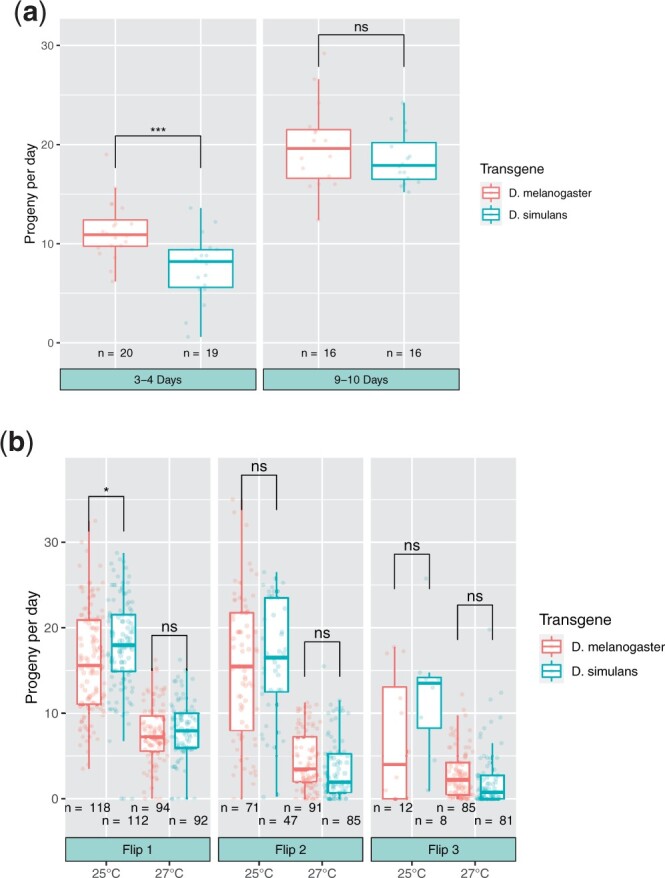
Comparison of fertility between *D. melanogastermh* mutant females carrying *mel-mh-Gfp* vs. *sim-mh-Gfp* transgenes (single *w mh^6^/w mh^31^; {mel-mh-Gfp, w^+^}attP40/+* vs . *w mh^6^/w mh^31^; {sim-mh-Gfp, w^+^}attP40/+*). Statistical significance between genotypes was tested by a 2 sample unpaired *t*-test: *** = 0.0001 < *P* ≤ 0.001; * = 0.01 < *P* ≤ 0.05; ns = *P* > 0.05 (not significant). a) Pilot experiment at room temperature (∼20–22°C). Females were collected as virgins and aged for either 3–4 days (left) or 9–10 days (right) prior to mating and progeny collection for 5 days. Progeny are reported as “per-day” to normalize with (b). b) Two large-scale experiments, performed at 25 and 27°C. Virgin females were aged for 3–5 days prior to mating. Crosses were flipped to fresh vials after 4 or 5 days, and again after an additional 4 or 5 days, for a total collection period of 12–13 days; the exact length of each flip was recorded and is accounted for in calculating the “progeny per day.” Note that vials were discarded if the parents died, which is why N goes down between flips (see *Materials and Methods*).

We constructed a transgene of *D. simulans mh* tagged with Gfp (called *sim-mh-Gfp*) to precisely match in structure the *D. melanogaster mh-Gfp* transgene of Tang *et al.* (2017) (Fig. 1; *Materials and Methods*). We transformed and integrated both transgenes into *D. melanogaster* at the same autosomal position. Western blots indicate that both transgenes express at similar levels (Fig. 2). We then crossed the transgenes into a *mh^6^* mutant background. Stable stocks were established, indicating that both transgenes can complement the sterility of *mh^6^*. To quantitatively compare the activity of these transgenes, we performed fertility assays of females *trans*-heterozygous for 2 *mh* null alleles and heterozygous for a *mh-Gfp* transgene; that is *w mh^6^/w mh^31^; {mel-mh-Gfp, w^+^}/+* compared to *w mh^6^/w mh^31^; {sim-mh-Gfp, w^+^}/+*. A small-scale pilot experiment found greater fertility among females carrying *mel-mh-Gfp*, but only among younger females (Fig. 3a). We then performed a more extensive experiment, examining fertility across a 12–13-day period at 2 different temperatures (Fig. 3b). No significant difference between the transgenic genotypes was observed at any time point, except for the first flip at 25°C, where females carrying the *mel-mh-Gfp* transgene had significantly fewer progeny than those carrying the *sim-mh-Gfp* transgene. We conclude that the *D. melanogaster* and *D. simulans* orthologs have not substantially diverged for the essential female fertility function of *mh*.

### *D. melanogaster mh* does not affect hybrid viability

Having established that *sim-mh-Gfp* is functional within *D. melanogaster*, we turned to the primary question motivating this study, of whether *mh* is a hybrid incompatibility gene. We tested this by comparing the *D. simulans* and *D. melanogaster mh* transgenes for their ability to modulate F1 hybrid female viability. Specifically, we sought to determine whether the addition of a *mh*^+^ transgene to the mothers of these hybrids would increase viability of their daughters. We transformed these transgenes into 2 different *attP* sites in *D. simulans*, with Western blots with an anti-Gfp antibody demonstrating similar protein levels for both transgenes (Fig. 2).

We then generated F1 interspecific hybrids by crossing *D. simulans* females heterozygous for either *mel-mh-Gfp* or *sim-mh-Gfp*, along with sibling females not carrying a transgene as controls, to wild-type *D. melanogaster* males (Table 2). The viability of the control F1 daughters not carrying a transgene was quite high, ranging from ∼47% to 69% relative to F1 sons, which limited our ability to detect substantial increases in the progeny of interspecific hybrids from mothers carrying a transgene. This relatively high rescue may reflect the fact that the transgenes were outcrossed to the *D. simulans w^501^* strain. We used this strain to be able to easily follow the transgenes in a *white* mutant background, but this strain is known to produce high hybrid viability in other crosses (Gérard and Presgraves 2012). Regardless, the results are opposite to our hypothesis. Both sets of crosses with the *sim-mh-Gfp* transgene showed increased female viability from transgenic mothers compared to control mothers, though only set D (with the 1048 insertion) was statistically significant. In contrast, crosses with the *mel-mh-Gfp* transgene showed essentially no differences in the relative viability of daughters between the transgenic and control genotypes. Within the resolution of our assay, we find no evidence suggesting that hybrid lethality results from the absence of *mel-mh* alleles in *D. simulans*.

**Table 2.** Testing *mh* transgenes for modulation of interspecific F1 hybrid viability

Set	Maternal genotype	# F1 hybrid daughters	# F1 hybrid sons	Ratio F1 daughters/sons	Relative ratio w/o and w transgene	*P*-Value
A	*w*	760	1106	0.69		
	*w; {mel-mh-Gfp}1029/+*	766	1105	0.69	0.99	0.895
B	*w*	781	1144	0.68		
	*w; {mel-mh-Gfp}1048/+*	830	1124	0.74	0.92	0.228
C	*w*	144	211	0.68		
	*w; {sim-mh-Gfp}1029/+*	84	88	0.95	0.71	0.0722
D	*w*	248	529	0.47		
	*w; {sim-mh-Gfp}1048/+*	461	693	0.67	0.70	0.000331

In the following descriptions, all flies are *D. simulans* unless noted otherwise. The designation *{mh-Gfp, w^+^}* refers to one of the 4 transgenic genotypes used in this table. The flies described in the “Maternal genotype” column were generated as follows: (1) *w^501^* virgin females were crossed to *y w/Y; {mh-Gfp, w^+^}* males. (2) *w^501^/y w; {mh-Gfp, w^+^}/+* virgin daughters were crossed to *w^501^/Y* males. (3) For each set*, y? w/w^501^; {mh-Gfp, w^+^}/+* and *y? w/w^501^* virgin daughters were separately collected. The females from step (3) were then mated to *D. melanogaster* Canton-S males. The full genotypes of the transgenes are noted in the *Materials and Methods*. *P*-Values were calculated using a 2 × 2 Chi-squared test.

## Discussion

The *mh* gene is interesting based on its unusual mutant phenotype of producing gynogenetic haploid embryos. It also displays a relatively high rate of coding sequence evolution, has experienced a recent duplication in *D. simulans*, and has a pseudogene fragment in both *D. melanogaster* and *D. simulans* that indicates a possible second duplication in their common ancestor. Our major motivation in this study derived from the association of mel-Mh with the X-linked 359-bp satellite DNA block that is present in *D. melanogaster* but absent in *D. simulans*. We used transgenic constructs to compare the activity of *mh* orthologs from *D. melanogaster* and *D. simulans* in the background of both species. We designed the *sim-mh-Gfp* transgene to be parallel in structure to a *mel-mh-Gfp* transgene previously shown to be functional. Our *sim-mh-Gfp* transgene complements *D. melanogaster mh* mutations, as evidenced by the ability to maintain a fertile *mh^6^; sim-mh-Gfp* stock.

In the course of this study, Brand and Levine (2022) independently published a study of the evolution of *mh* between *D. melanogaster* and *D. simulans*. They compared *mh* ortholog function by replacing the endogenous *D. melanogaster mh* locus with the *D. simulans* ortholog. They found that females of this replacement line have reduced fertility compared to the *D. melanogaster mh* control line and further found that this reduced fertility is dependent on an intact *Zhr*^+^ locus, which contains a large block of the 359-bp satellite. Below we summarize our results and compare them to those of Brand and Levine where appropriate.

### Similar fertility function of *mel-mh* and *sim-mh*

We observed some reduction of fertility in *D. melanogaster mh* mutants carrying *sim-mh-Gfp* compared to *mel-mh-Gfp* controls in one of the 2 small scale initial experiments (Fig. 3a). However, follow-up experiments at much larger scale failed to find any reduction (with 1 out of the 6 comparisons showing a modest but significant increase in fertility of *sim-mh-Gfp* relative to *mel-mh-Gfp* females; Fig. 3b). We conclude that *D. melanogaster* and *D. simulans mh* orthologs are largely interchangeable for female fertility.

This contrasts with the result of Brand and Levine (2022), who report a more than 2-fold mean reduction in fertility of *D. melanogaster* females carrying *sim-mh* compared to *mel-mh*. One possible explanation for these differences is that the fertility of *mh* genotypes are inherently variable due to environmental (or other) variation, which is plausible for any genotype that may be subfertile but not completely sterile. Another possibility may be differences in experimental design of the fertility assays. We assayed females individually while Brand and Levine analyzed fertility in broods of 4 females. If a female (and/or the males they were mating with) died during the course of our experiment we could exclude that vial, while in the Brand and Levine design individual deaths may not have been recorded and would reduce the brood size and thus presumably the progeny count. We saw very high rates of death in one of our experiments (at 25°C) that varied by genotype: *mel-mh-Gfp* crosses dropped from 118 to 71 from flip 1to 2, while for *sim-mh-Gfp* the drop was much greater, from 112 to 47.

There are also significant differences in experimental design of the gene replacements between the 2 studies. Here, we have used transgenic constructs integrated into an autosomal site and crossed into *mh* null-allele backgrounds to “replace” *mh* and compare *mel-mh* and *sim-mh*. We designed our *sim-mh-Gfp* transgene to match a previously described *mel-mh-Gfp* transgene that was shown to provide wild type *mh* function (Tang *et al.* 2017). This introduces a potential position effect as *mh* is now in an autosomal location. It is also possible that *sim-mh-Gfp* does not express properly in a *D. melanogaster* background since it has its endogenous regulatory regions. We did, however, see robust protein expression from our transgenes. Brand and Levine (2022) used a CRISPR replacement strategy, replacing the endogenous *mh* locus in *D. melanogaster* with FLAG-tagged and codon-optimized *mel-mh* and *sim-mh* coding sequences. This means that the *sim-mh* allele is synthetic in the sense that the synonymous sites in the transgene are not native to *D. simulans*. But again, Western blots suggest that it is fully expressed. Among other differences in the 2 studies is that our fertility assays were done with 1 functional copy of *mh* in the mothers (that is heterozygous for the *mh^+^* transgene), while the Brand and Levine (2022) design has 2 functional copies (that is, homozygous for the replaced locus). Brand and Levine (2022) found that *D. melanogaster* females with 1 *mel-mh* and 1 *sim-mh* allele are fully fertile, and other experiments support their conclusion that deleterious effects of *sim-mh* on fertility and ovarian morphology are dose dependent.

### Lack of effect of *mel-mh* on hybrid viability

We found no evidence that the absence of *mel-mh* contributes to hybrid female lethality. In crosses of *D. simulans* females to *D. melanogaster* males, *D. simulans* mothers carrying a *mel-mh-Gfp* transgene produced the same ratio of female hybrids compared to control crosses without the transgene. Repeating our experiments in *D. simulans* genetic backgrounds that have a lower baseline of hybrid female viability might therefore reveal more subtle effects that we could not detect. We also note that the hybrids contain both endogenous expressed *sim-mh* as well as *sim-mh-Gfp* or *mel-mh-Gfp* expressed from the transgene. It remains possible that potential effects of *mel-mh* in hybrids could be masked by the presence of the endogenous *sim-mh*. Surprisingly, we did observe a moderate effect of increased hybrid female viability produced by mothers that carried the *sim-mh-Gfp* transgene. This finding suggests that increased *mh* dosage may reduce mis-segregation of the 359-bp satellite in hybrids but that such effects are not dependent on the *mel-mh* ortholog that has coevolved with the *D. melanogaster* 359-bp satellite block.

### Whither the *D. simulans* maternal effect?

The identity of the *D. simulans* genes that are interacting with the *D. melanogaster* 359-bp satellite to cause hybrid lethality remain unknown. One approach would be to map the alleles that distinguish the rescuing *D. simulans mhr* strain from other *D. simulans* strains that do not rescue hybrid lethality. Mapping efforts could also be extended to include other *D. simulans* strains that produce high F1 hybrid daughter viability (Gérard and Presgraves 2012). Another approach could test alternative hypotheses, such as that hybrid lethality results from a lack in *D. simulans* of small RNAs derived from the 359-bp satellite (Ferree and Barbash 2007). This would require developing a transgenic expression system in *D. simulans* that drives the deposition of such small RNAs into the developing egg. If this turned out to represent the mechanistic basis of hybrid female lethality, it remains of high interest to understand why there is such substantial interstrain variation in *D. simulans* for this hybrid lethality.

## Data availability

Strains and plasmids are available upon request. The authors affirm that all data necessary for confirming the conclusions of the article are present within the article, figures, and tables.

Supplemental material is available at *G3* online.

## Supplementary Material

jkac177_Supplementary_Table_S1

jkac177_Supplementary_File_1
