# Supplementary material for: Testing the Drosophila maternal haploid gene for functional divergence and a role in hybrid incompatibility
Source: G3 (Bethesda). 2022 Jul 25;12(9):jkac177. doi: 10.1093/g3journal/jkac177 (PMC9434238; doi:10.1093/g3journal/jkac177)
Supplement: jkac177_Supplementary_Table_S1 [file jkac177_supplementary_table_s1.docx]

Table S1. Oligonucleotide sequences

| **Oligo #** | **Oligo Sequence (all 5'-3')** |
| --- | --- |
| 498 | GGAGTACAACTACAACAGCC |
| 502 | aaacccgcggccgcATGCCCGCCGTGACCGTC |
| 503 | aaacccgcggccgcGATGTAGGTCACGGTCTCG |
| 788 | CACAGGAAACAGCTATGACCATGA |
| 823 | CGTTGTAAAACGACGGCCAGT |
| 2016 | CATGCTAGCGGCCGCGGACATGGCCACGTAATAAGTGTG |
| 2017 | ACTACGATCGGTTGTTGGTTGGCACACC |
| 2018 | AACCAACAACCGATCGTAGTGCCCCAAC |
| 2019 | TACGTGGCCATAAGATACATTGATGAGTTTGGACAAAC |
| 2020 | ATGTATCTTATGGCCACGTAATAAGTGTG |
| 2021 | CTTATGCATGGAGATCTTTAGTTGTTGGTTGGCACACC |
| 2078 | CATGAAGCGTCTTCTCGCTG |
| 2079 | CCAACCTTCTAAAAGACTTGTC |
| 2080 | GGGATTCGCATTAGAATTGT |
| 2081 | CGCAAACCACTGTCGTTAAA |
| 2082 | CTTACCAAGAGCGTCTCCA |
| 2083 | GTTCATTGCCTCCGCCACT |
| 2084 | TAAACGATGGCGGTTCCAA |
| 2099 | accggtctagaGGCTTGCTTTAAAAGCTGATTATC |
| 2100 | accggtctaGAAGGGCAACATGACGACCAGC |
| 2101 | GGAGAGGACACCCTGCTGAAG |

Lower case indicates restriction sites added for subsequent cloning.
